# Neuroinflammation mechanism underlying neuropathic pain: the role of mesenchymal stem cell in neuroglia

**DOI:** 10.3934/Neuroscience.2024015

**Published:** 2024-07-12

**Authors:** Ida Ayu Sri Wijayanti, I Made Oka Adnyana, I Putu Eka Widyadharma, I Gede Eka Wiratnaya, Tjokorda Gde Bagus Mahadewa, I Nyoman Mantik Astawa

**Affiliations:** 1 Doctoral Program in Medical Sciences, Faculty of Medicine, Universitas Udayana, Bali, Indonesia 80232; 2 Department of Neurology, Faculty of Medicine, Universitas Udayana, Bali, Indonesia 80232; 3 Department of Orthopedics and Traumatology, Faculty of Medicine, Universitas Udayana, Bali, Indonesia 80232; 4 Department of Neurosurgery, Faculty of Medicine, Universitas Udayana, Bali, Indonesia 80232; 5 Department of Pathobiology, Faculty of Veterinary Medicine, Universitas Udayana, Bali, Indonesia 80232

**Keywords:** neuroinflammation, neuropathic pain, mesenchymal stem cell, neuroglia

## Abstract

Pain is an essential aspect of the body's physiological response to unpleasant noxious stimuli from either external sustained injuries or an internal disease condition that occurs within the body. Generally, pain is temporary. However, in patients with neuropathic pain, the experienced pain is persistent and uncontrollable, with an unsatisfactory treatment effectiveness. The activation of the immune system is a crucial factor in both central and peripheral neuropathic pain. The immune response plays an important role in the progression of the stages of neuropathic pain, and acts not only as pain mediators, but also produce analgesic molecules. Neuropathic pain has long been described as a result of dysfunctional nerve activities. However, there is substantial evidence indicating that the regulation of hyperalgesia is mediated by astrocytes and microglia activation. Mesenchymal stem cells currently hold an optimal potential in managing pain, as they can migrate to damaged tissues and have a robust immunosuppressive role for autologous or heterologous transplantation. Moreover, mesenchymal stem cells revealed their immunomodulatory capabilities by secreting growth factors and cytokines through direct cell interactions. The main idea underlying the use of mesenchymal stem cells in pain management is that these cells can replace damaged nerve cells by releasing neurotrophic factors. This property makes them the perfect option to modulate and treat neuropathic pain, which is notoriously difficult to treat.

## 1 Introduction

According to the International Association for the Study of Pain (IASP), neuropathic pain is directly caused by a lesion or disease that affects the peripheral or central somatosensory system. About 20–25% of chronic pain cases are characterized by neuropathic pain, which affects the patient's physical and mental health [1]–[3]. Neuropathic pain can manifest as spontaneous pain sensations such as paroxysmal pain or superficial pain, an increased response to painful stimuli (hyperalgesia), allodynia (a pain response due to non-noxious stimuli), or temporal summation (a heightened pain sensation due to recognized and repetitive stimuli) [4],[5].

Neuropathic pain progresses into chronic pain, which is a consequence of psychological, biological, and social factors, and requires a multifactorial approach for evaluation and management. The current management of neuropathic pain primarily focuses on pharmacological therapies. Therefore, it remains a significant challenge, making it crucial to seek new therapy strategies with long-term effects and more optimal outcomes [2],[6],[7].

## 2 Review

### 2.1. Neuropathic pain mechanism

Neuropathic pain is induced by various mechanical damages and through a complex cascade because of a nerve injury, which manifests as heterotopic pain, hyperalgesia, burning sensations, tingling, and spontaneous pain. Changes in the activity of various genes due to axon transection can lead to differences in the composition of neurotrophic factors at the injury site, where the sensory axons become generators of pathological ectopic pulsations [2],[8]. An increase in the sodium channel density can alter the nerve sensitivity and increase damage to a number of axon membrane receptors in adrenergic neurons. Due to peripheral nerve injuries, abnormal sympathetic nerve structures form around the dorsal root ganglion (DRG) neurons, resulting in symptoms of sympathetic nerve pain that arise from the peripheral nerve injury [8],[9].

The second-order neurons in the spinal cord transmit nociceptive impulses to the thalamus via the ascending pathway, known as the spinothalamic tract, where the thalamus serves as a relay station to higher cortical centers. These centers include the anterior cingulate cortex (involved in anxiety, the anticipation of pain, attention to pain, and the motor response), the insular cortex (responsible for discriminative sensory and affective aspects of pain contributing to negative emotional responses and pain-related behaviors), the prefrontal cortex (important for sensory integration, decision-making, memory, and attention processes related to painful stimuli), the primary and secondary somatosensory cortex (involved in localizing and interpreting noxious stimuli), and the amygdala and hippocampus (limbic system), which are involved in forming and storing memories associated with emotional events, affect, arousal, attention to pain, and the learning processes. The limbic system might also be responsible for the fear that accompanies painful sensations. Pain can be understood as a multidimensional experience, where psychosocial factors such as depression, somatization, poor coping abilities, social stressors, and tendencies toward a negative job satisfaction can predict the development of chronic pain after an acute episode [10],[11].

### 2.2. Peripheral neuropathic pain mechanism

In normal conditions, the activation of primary afferent neurons occurs upon stimulation. After a nerve injury, this is followed by neuroinflammatory processes and tissue repair, leading to a state of hyperexcitability known as peripheral sensitization. This condition often resolves as part of the healing and subsiding inflammation. Primary afferent neurons undergo long-lasting alterations if nociception is sustained by recurring stimuli from the injury. Several factors contribute to the mechanism of peripheral sensitization. Inflammatory mediators such as calcitonin gene-related peptide and substance P, which are released from nociceptive terminals, increase the vascular permeability, causing a local edema and stimulating the release of other substances due to injury, such as prostaglandins, bradykinin, growth factors, and cytokines. These substances induce sensitization and stimulate nociceptors, resulting in a lower firing threshold and ectopic discharge. Ectopic discharge can cause spontaneous pain and may originate from the DRG, other locations along the damaged nerve, or even adjacent normal nerve fibers, which can result in spontaneous pain. The process by which normal and neighboring nerve fibers become excited due to the non-synaptic ephaptic crosstalk is known as ephaptic transmission [11],[12].

### 2.3. Central neuropathic pain mechanism

Sensitization within the central nervous system (CNS) refers to functional synaptic plasticity that depends on the activity and leads to a pain hypersensitivity. This plasticity is associated with activity in the dorsal horn neurons, which receive inputs from nociceptive C-fiber neurons. Interestingly, afferent nociceptors that innervate muscles or joints produce a longer-lasting central sensitization than those that innervate the skin. The primary mechanism related to inducing acute activity-dependent central sensitization involves N-methyl-D-aspartate (NMDA) receptors, which are associated with glutamate and its receptors [13].

Components within the spine that are crucial in neuropathic pain mechanisms includes synaptic plasticity manifested as temporal and spatial summation (enhanced neuron response to repeated and simultaneous noxious stimulation). The other elements involved encompassing the proliferation of nociceptors and second-order neurons, as well as the heightened excitability of neurons in the pain pathway that relay signals to the supraspinal regions. These neuroplasticity alterations occur throughout the nociceptive pathway from the spinal cord to the brain.

At a cellular level, the transmission of nociceptive signals within the CNS is regulated by cellular and intracellular elements, including ion channels (Na^+^, Ca^++^, K^+^), ionotropic and metabotropic receptors such as glutamatergic, gamma-aminobutyric acid (GABA), serotonin, adrenergic, neurokinin, and vanilloid receptors, inflammatory cytokines released from activated glial cells, nerve growth factors, and intracellular regulators (protein kinases and transcription factors) [11],[13].

After a nerve injury, the loss of inhibition occurs due to disruptions in GABA's production and release mechanisms. This disruption leads to intracellular homeostatic imbalances due to the reduced activity of the K^+^-Cl cotransporters and/or the increased activity of the Na^+^ K^+^-Cl- cotransporters, which results in elevated Cl- levels and apoptosis of the spinal inhibitory interneurons. The loss of inhibitory control has been shown to trigger tactile allodynia and hyperalgesia, causing structural changes in Aβ fibers, thereby increasing the transmission of noxious stimuli to second-order neurons in the dorsal horn (under normal conditions, Aβ fibers transmit non-noxious stimuli). Following a nerve injury, the DRG exhibits a decreased expression of the µ-opioid receptors, and second-order spinal neurons become less responsive to opioids. Conversely, the inflammatory process can lead to an increase in the number and affinity of opioid receptors, which enhances the opioid effectiveness. Hence, chronic neuropathic pain requires higher opioid doses compared to acute or chronic nociceptive pain conditions [11],[14].

Prolonged pain activation triggers the activation of astrocytes and microglia by neuronal signals, including substance P, glutamate, and fractalkine. The activation of glial cells by these substances leads to the release of mediators that subsequently act on both glial cells and other neurons [15].

### 2.4. The role of immune cells in neuropathic pain

Immune system activation is a crucial factor in both central and peripheral neuropathic pain. Neuroimmune interactions in pain mechanisms are bidirectional. Immune cells release various cytokines, lipids, and growth factors that act on nociceptors and neurons in the CNS to sensitize pain signal pathways. Subsequently, nociceptors actively release neuropeptides from peripheral nerve terminals to modulate the activity of innate and adaptive immune cells [18].

The accumulation of endoneurial neutrophils represents the initial stage in the pathogenesis of hyperalgesia in a peripheral nerve injury. Neuroimmune interactions result from peripheral nerve injuries and are crucial in the development of neuropathic pain. Several studies suggest that macrophages play a role in the pathogenesis of allodynia and hyperalgesia. Macrophages are significantly involved in Wallerian degeneration, which is a characteristic of chronic neuropathic pain marked by the release of pro-inflammatory cytokines during nerve damage. Moreover, Wallerian degeneration is a critical factor in the pathogenesis of hyperalgesia. Following nerve damage, macrophages surround the area of Wallerian degeneration. Besides macrophages, T cells also participate in chronic constriction injury (CCI), demonstrating the involvement of macrophages, lymphocytes, and satellite cells in facilitating the immune response in the DRG [19],[20].

Various immune cells release mediators that act on nociceptor terminals to induce peripheral sensitization. Within the CNS, microglia, astrocytes, and T-cells modulate neurotransmission and pain circuits in the spinal cord to promote central sensitization. During acute inflammation, the emergence of pain aligns with the immune response activation and tends to heal as the inflammatory process resolves. In chronic conditions, persistent immune cells mediate long-lasting pain [18].

Pain accompanied by nerve damage can be mediated by cytotoxic natural killer cells. Nerve damage triggers a cascade due to an inflammatory response. After nerve damage occurs, glial cells are activated, releasing cytokines and chemokines, followed by the involvement of neutrophils. Neutrophils are typically the first immune cells to respond in the case of tissue damage. Monocyte-macrophage infiltration into nerve damage occurs over several hours to days. Some studies have indicated a significant increase in the number of T-cells between 7–28 days after nerve cell damage [17],[21].

The primary signaling molecules in immune interactions are cytokines. Cytokine receptors have been found in the CNS on pseudounipolar neurons, secondary nociceptive neurons, astrocytes, and microglia. The peripheral effects of cytokines on the CNS are restricted by the BBB, while the permeability is controlled by higher parts of the CNS and humoral factors. Activated glial cells are the primary source of cytokines in the CNS. Macrophages that have breached the BBB also play a role in neuroinflammation and serve as a source of cytokines in the CNS [22].

The inflammatory process is highly crucial in developing neuropathic pain, not only by causing various changes in the extracellular environment, but also by inducing intracellular alterations. Several components play a role in this inflammatory process, including the accumulation and activation of inflammatory cytokines, chemokines, and prostaglandins, the modulation of extracellular proteins, changes in transmembrane receptor expression, immune cell infiltration, and alterations in intracellular modulation through ion channel activity and receptor signaling. While the inflammatory response has been observed in most body tissues, certain cell types are explicitly found in the CNS: neurons, microglia, astrocytes, and oligodendrocytes [23].

Factors such as pro-inflammatory cytokines, oxidative stress, and free radicals induce reactivity in astrocytes. This astrocyte reactivity is initiated through the Nuclear Factor kappa-B (NFκB) pathway. Astrocyte activation and the release of IL-1β trigger hyperalgesia. These cytokines and chemokines induce and sustain persistent pain. Astrocyte activation has been investigated concerning the pathogenesis of pain, which involves the release of various pro-inflammatory and pro-algesic mediators such as TNF-α, IL-1β, and IL-6. Active astrocytes can initiate and maintain chronic pain events by releasing various components. Moreover, these active astrocytes can regulate emotions in several brain regions, including the primary somatosensory cortex, the anterior cingulate cortex, the hippocampus, prefrontal areas, and the medial cortex, which is associated with emotional changes during chronic pain [25],[29].

### 2.5. The role of glial cells in neuropathic pain

In cases of excessive pain activation, astrocytes and microglia are triggered by neuronal signals, including substance P, glutamate, and fractalkine. The activation of glial cells by these substances leads to the release of mediators that subsequently act on both glial cells and other neurons [15].

Glial cells make up nearly 70% of all cells in the CNS, and under normal conditions, resting microglia are fewer in number. The CNS is comprise of two types of glial cells: macroglia and microglia, which consists of astrocytes, oligodendrocytes, and satellite cells, including Bergmann cells and Müller cells. Recent studies have highlighted the vital role of glial cells in maintaining CNS homeostasis. Meanwhile, glial cells in the peripheral nervous system (PNS) consist of Schwann cells in the peripheral nerves, satellite glial cells (SGCs) in the DRG, and trigeminal ganglion and enteric glial cells. Glial cells do not transmit nerve impulses like neurons, but collectively play a crucial role in neurotransmitter synthesis and release. They play protective roles in forming the BBB, developing the myelin sheath, contributing to neuron nutrition, and are involved in cellular defence mechanisms. Injuries to the peripheral nerves, CNS hypoxia, and neuronal degradation are associated with the activation of Schwann cells around damaged nerves, satellite cells in the DRG, astrocytes and microglia at the spinal level [15]. After a nerve injury, microglial activation occurs in the dorsal horn of the spinal cord within the first 24 hours. In contrast, astrocytes activation is shown to occur later, around the third-day post-injury. Microglial activation lasts no more than 3 months and then decreases in number, which correlates with the onset of neuropathic pain symptoms such as allodynia or hyperalgesia. The role of glial cell activation in synaptic transmission is based on receptors, ion channels, transporters, and similar intracellular signaling pathways found on the surface of neurons and glial cells. Damage to the spinal cord and peripheral nerves triggers the physiological and morphological activation of glial cells, especially astrocytes and microglia [15],[24].

Astrocytes are the most abundant type of macroglia cells found in the CNS. Through various transport proteins, astrocytes regulate homeostasis in the CNS by controlling the levels of extracellular ions, proteins, and neurotransmitters in their surrounding environment. Astrocytes activation leads to morphological changes, such as hypertrophy and an increased production of glial fibrillary acidic protein (GFAP), and functionally, it enhances the production of other substances such as pro-inflammatory factors [15]. Through the interaction between astrocytes and microglia, astrocytes can modulate the microglia phenotype and phagocytic activity. Astrocytes play a crucial role in the induction and persistence of pain. When damage or harmful stimuli such as nerve inflammation and ischemia occur, astrocytes undergo morphological and functional changes known as reactive astrogliosis [21],[25].

The pain syndrome is also associated with glial cell activation, leading to increased glial cell markers such as IBA1 and GFAP, which results in morphological changes. During reactive astrogliosis, naïve astrocytes undergo hypertrophy and increased GFAP levels. Subsequently, reactive astrocytes proliferate, leading to the formation of scar-forming astrocytes. Both forms of astrocytes exhibit higher levels of protein markers, including GFAP, nestin, β-catenin, and N-cadherin. Additionally, this process involves the synthesis and release of glial cell mediators such as growth factors, cytokines, chemokines, and proteases, which can heighten the pain sensitivity. Increased GFAP is a biomarker for reactive astrocytes [25].

GFAP is a primary intermediate filament of astrocytes that plays a crucial role in maintaining the integrity and motility of astrocytes. It contributes to the architecture of the white matter, myelination, and the integrity of the BBB. GFAP is a highly brain-specific protein because the relevant extracerebral sources of this protein have not been identified, even though it is also expressed in the glial cells of the peripheral nervous and enteric systems. It is postulated that GFAP could enter the bloodstream from brain tissue due to two factors: the loss of astrocytic structural integrity through necrosis and/or mechanical disruption and the breakdown of the BBB [30],[31].

**Figure 1.**
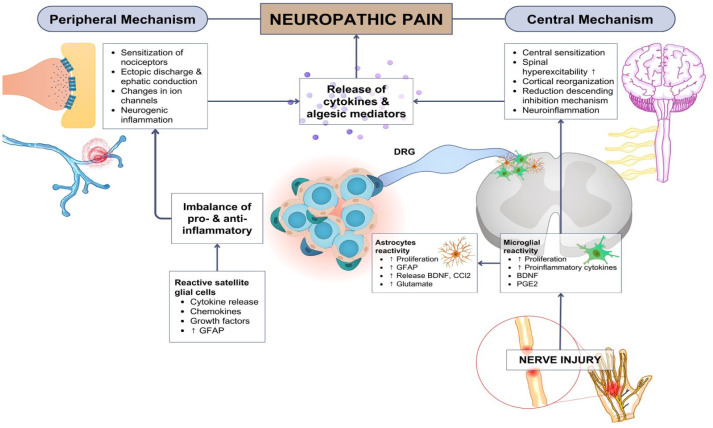
Neuropathic pain: the involvement of glial cells and neuroinflammation.

Injuries to the peripheral or central nervous systems generate maladaptive alterations in neurons along the nociceptive pathway, which can lead to neuropathic pain. Microglia are activated after nerve injuries. Microglia become activated by injury released cytokines and proinflammatory factors, which stimulate the activation of astrocytes; this process involves several downstream signaling pathways, such as neuroinflammation.

Several studies have indicated that the process of neuroinflammation leads to the activation of microglia and astrocytes, which play a crucial role in the mechanism of neuropathic pain. Astrocytes are more involved in chronic pain conditions and synapses after nerve injuries compared to microglia. Microglia play a more significant role of initiating neuropathic pain, whereas astrocytes are more implicated in persistent neuropathic pain conditions. Neuroinflammation begins as a response to various exogenous and endogenous sources, including various pathogens, nerve injuries, and toxic compounds. This condition is characterized by glial cell activation, the release of various inflammatory molecules, and an increased BBB permeability. Neuroinflammation is initiated by the activation of microglia, which are resident immune cells in the CNS. Under steady-state conditions, microglia remain in a resting state through interactions between cell surfaces and soluble factors in their surrounding cells. Microglial activation occurs after exposure to PAMPs and/or endogenous DAMPs and the loss of immune suppression signals. Microglial activation leads to the production of pro-inflammatory cytokines and chemokines, including IL-1β, IL-6, and TNF-α, which act as pro-inflammatory mediators. Additionally, these chemokines are implicated in inflammatory factors in pain, cancer-related pain, and neuropathic pain. Spinal microglia respond to extracellular stimuli by facilitating signal transduction through intracellular cascades such as mitogen-activated protein kinase, p38, and protein kinases regulated by extracellular signals [15]–[17].

### 2.6. Mesenchymal stem cell

Transplantation of mesenchymal stem cell (MSCs) is now frequently employed as an alternative for regenerative therapy in promising pain management cases, especially treating osteoarthritis, neuropathic pain, and intractable musculoskeletal pain that does not respond well to conventional therapy. Stem cell therapy has the ability to differentiate into various cell phenotypes. MSCs are a source of totipotent cells that can replace damaged or lost parts of neuronal cells. Furthermore, MSCs possess tropic factors for injured nerves. They have the ability of transdifferentiation, which refers to their capacity to become cells outside of their differentiation pathway. MSCs actively contribute to their surrounding environment by secreting cytokines, growth factors, and extracellular matrix molecules that play crucial roles, both for themselves (autocrine) and for the neighboring cells (paracrine) [5],[32]. MSCs can repair nerve damage, not just to alleviate symptoms caused by neuropathic pain syndrome [33],[34].

MSCs can enhance the survival of motor and sensory nerves, improve motor function, induce neurogenesis and axonal growth, increase myelin formation, and reduce pain through the regulation of GDNF. Additionally, Al-Massri et al. reported that MSCs can reverse the decreased expression of nerve growth factor (NGF) in patients with nerve injuries and regulate the neuroprotective effects of NGF by enhancing axonal growth and nerve survival [35].

The main principle behind applying stem cells in neuropathic pain is their ability to provide a cellular source to replace injured nerve cells and release neurotrophic factors, including epidermal growth factor, BDNF, NT-3, Ciliary Neurotrophic Factor (CNTF), basic Fibroblast Growth Factor (bFGF/FGF-2), hepatocyte growth factor, and vascular endothelial growth factor (VEGF). Currently, various stem cells such as bone marrow mesenchymal stem cells, human amniotic fluid-derived mesenchymal stem cells (hAFMSCs), adipose-derived stem cells (ADSCs), and GABAergic intermediate neuron progenitor cells have strong therapeutic potentials to manage neuropathic pain with promising outcome [34],[36].

Adipose-derived Mesenchymal Stem Cells (ADMSCs) are multipotent, which means that they can differentiate into various cell types. They can expedite the improvement of allodynia, which is a manifestation of central sensitization, through their multi-differentiation potential, their ability to renew cells by replacing damaged nerve cells, their ability to provide various nutritional factors to lesion sites, and their immunomodulatory capacities. The effectiveness of stem cell administration in neuropathic pain conditions is also associated with the interaction between stem cells and resident cells within the micro-environment. MSCs have the potential to inhibit degenerative processes, impede apoptosis, and enhance the nerve's resilience and repair capabilities in both the peripheral and central nervous systems through the release of various neurotrophic factors.

There are several studies that have been conducted regarding the role of MSCs. Its unique activities include peripheral and spinal mechanisms. The detail of MSCs' unique properties are further displayed in Table 1.

**Table 1.** The role of MSCs in peripheral mechanism and spinal mechanism.

**Author (Year)**	**Model of Neuropathic Pain**	**Cell Type**	**Peripheral Mechanism**
Chen et al. (2015) [36]	CCI (mice)	BMSCs	Inhibited expression of ATF3 in DRG neurons induced by CCI. Reversed the downregulation of CGRP and IB4 staining in central axon terminals of DRG neurons and spinal dorsal horn caused by CCI.
Chiang et al. (2016) [37]	CCI (rats)	AFMSCs	Increased the expression of IL-1 β, CD68, and TNF-α, and decreased the expression of S100 and neurofilament in the injured nerve.
Mert et al. (2017) [38]	CCI (rats)	ADMSCs	Decreased IL-1β and IL-6 in the sciatic nerve and increased IL-10 expression.
Xie et al. (2019) [39]	CCI (mice)	BMSCs	Inhibited CCI-induced p-ERK1/2 expression in the DRGs, and increased the amount of TGF-β1 protein in the DRGs. TGF-β1 attenuated NP through inhibition of p-ERK1/2.
Al-Massri et al. (2019) [40]	Paclitaxel- induced neuropathy	BMSCs	Increased expression of NGF in the sciatic nerve, reversed the increase of NF-κBp65, TNF-α, and IL-6 caused by CCI.
Chen et al. (2015) [41]	CCI (mice)	BMSCs	Released TGF-β, regulated the excitatory synaptic transmission of spinal cord neurons, and reduced the increase in neuronal excitability after nerve injury, thus resisting the development of central sensitization.
Guo et al. (2016) [42]	SNL (rats)	BMSCs	Inhibited the phosphorylation of GluN2A in RVM, reduced the expression of PKCG, inhibited the expression of NMDA receptors, thus resisting the development of central sensitization.
Forouzanfar et al. (2018) [43]	CCI (rats)	BMSCs	Decreased the increase of GFAP expression in rat spinal cord induced by CCI, reduced the expression of TGF-α, and reduced the apoptosis of tissue cells.
Huang et al. (2018) [44]	Noncompressive disk herniation (rats)	BMSCs	Decreased the mRNA and protein expression of TNF-α and IL-1β, upregulated the expression of TGF-β, and reduced the activation of microglia in the dorsal horn of the spinal cord.
Romero et al. (2020) [45]	CCI (rats)	BMSCs	Reduced the activation of spinal microglia, apoptosis, and autophagy of spinal cord cells.
Watanabe et al. (2015) [46]	CCI (mice)	BMSCs	Decreased the activation of p-p38MAPK and pERK1/2 in microglia induced by SCI, and the expression of CREB and PKC-c in injured and surrounding dorsal horn neurons.
Teng et al. (2019) [47]	CCI (rats)	BMSCs	Inhibited the expression of P2X4R in spinal microglia but did not affect the activation of microglia induced by CCD.

### 2.7. The role of stem cells in neuropathic pain mechanism

#### 2.7.1. Anti-inflammatory regulation

Stem cells are immunosuppressive and play a role in anti-inflammatory mechanisms by regulating and releasing various immunomodulatory, angiogenic, and cell-nutrient factors. They decrease harmful immune responses and inflammation while repairing tissue damage in other microenvironments. Research has shown that stem cells can treat various diseases, such as heart failure and lung fibrosis, through anti-inflammatory effects. Currently, several studies on managing neuropathic pain using stem cells focus on their anti-inflammatory effects. In a study by Mert et al., therapy using adipose stem cells significantly reduced pro-inflammatory factors such as IL-1β and IL-6 induced by chronic constriction nerve injury (CCI) in the Sciatic Nerve and increased IL-10, which is an anti-inflammatory marker. These results might be due to the interaction between stem cells and monocytes/macrophages, as stem cells encourage macrophage polarization into an anti-inflammatory phenotype. Stem cells act as anti-inflammatories through the mitogen-activated protein kinase (MAPK) pathway. After a nerve injury, signals from the damaged axons activate the extracellular MAPK signal pathway related to signals in Schwann cells, which is one of the initial events that trigger the expression of inflammatory mediators and activate immune cells in the injured nerve [47].

#### 2.7.2. Neuroprotection and Increased Regeneration of Axonal Myelin

Nerve damage induces abnormal neuron excitation, leading to nerve fiber degeneration, altering gene expression pathways, and causing ectopic discharge. Spontaneous ectopic activity in nerve endings or axons is crucial in spontaneous pain occurrence and drives abnormal pain responses. Activating Transcription Factor 3 (ATF3) is a marker for nerve injury in the DRG. Chen et al. proved that ATF3 immunoreactivity in the L4-L5 DRG neurons significantly increased by up to 40% in the CCI model; four days after intrathecal BMSC administration, there was a 14% inhibition in ATF3 expression. Additionally, a nerve injury reduces the regulation of neuropeptides, such as calcitonin gene-related peptide (CGRP) in peptidergic neurons and isolectinB4 (IB4) bound by nonpeptidergic neurons in the DRG. Stem cells decrease CGRP and IB4 regulation in CCI-induced DRG neurons and protect against axonal injury, indicating that stem cell therapies reduce persistent nerve damage [48],[49].

GDNF is a neurotrophic factor that alleviates neuropathic pain in animal models and acts as a neuroprotector. Several studies used GDNF and its receptors as targets to develop new analgesic therapies. Stem cells enhance the survival of motor and sensory nerves, improve motor function, induce neurogenesis and axon growth, increase myelin formation, and reduce pain through GDNF regulation. Additionally, Al-Massri et al. reported that stem cells can reverse the decline in NGF expression in patients with nerve injuries and regulate NGF's neuroprotective effects by enhancing axonal growth and nerve survival [48].

VEGF is a crucial regulator in nerve regeneration, and is a supporting factor that enhances nerve regeneration through angiogenesis, neuron nutrition, and neuroprotection, thereby restoring nerve function. Stem cell therapy plays a unique balancing role in VEGF regulation; however, the specific interaction between VEGF and stem cells requires further research [48].

VEGF-A plays a crucial role in the nervous system by enhancing cell endurance, increasing peripheral nerve density, and improving pain conditions. Targeting therapies towards VEGF is expected to reduce pain behaviors in various animal models. Meanwhile, several studies have reported the potential use of VEGF-expressing stem cells as one of the options for pain management [49].

#### 2.7.3. Decrease and inhibit the central sensitization process

Marked by increased nerve activation, central sensitization is considered one of the most crucial mechanisms underlying neuropathic pain. After nerve injury, there is an increased release of the amino acid glutamate in the DRG of the spinal cord, and the N-methyl-D-aspartate receptor (NMDAR) continues to be activated to maintain afferent nerve transmission to the brain. Long-term stimulation due to chronic nerve injury leads to the regulation of NMDAR and triggers central sensitization. Studies with animal models have shown that inhibiting NMDAR reduces neuropathic pain. Specific NMDAR antagonists have been intermittently used in managing neuropathic pain. Guo et al. intravenously injected bone marrow stem cells (BMSCs) in rats with tendon and spinal nerve ligation; they found that BMSCs could inhibit the expression of NMDA receptors and protect against glutamate excitotoxicity, thereby alleviating mechanical hyperalgesia after spinal cord injury in rats and providing analgesic effects [47],[50].

#### 2.7.4. Inhibit the Activity of Glial Cells

Many studies have indicated that stem cells' analgesic and long-term therapeutic effects are closely associated with the role of glial cells. Glial cells constitute about 70% of all cells in the CNS and play a crucial role in maintaining homeostasis in the body.. Literature suggests that microglia are activated within 24 hours after nerve injury; astrocytes are activated immediately after nerve injury, and their activation lasts up to 12 weeks [16],[51]. The subsequent cytokine release from astrocytes and microglia induces a series of cellular responses, such as an increased regulation of glucocorticoid and glutamate receptors, leading to excitation in the spinal cord and neuroplasticity processes. This is closely associated with neuropathic pain symptoms such as pain hypersensitivity [47].

Stem cells effectively inhibit the activation of glial cells. For instance, there is an increase in GFAP expression in the spinal dorsal ganglion of mice with CCI conditions. In a study by Forouzanfar et al., an intravenous administration of ADMSCs reduced GFAP expression by up to 1.2 times compared to the control [52]. On the other hand, Huang et al. reported that an intrathecal administration of BMSCs reduced the microglial activity in the dorsal horn of the spinal cord in rats with non-compressive disc herniation. Moreover, this administration improved the behavior of hyperalgesia associated with radicular pain by reducing the production of inflammatory cytokines generated by spinal microglia activation [53]. Another study found that in post-injury conditions, microglial cells marked with anti-Iba1 antibodies showed an expression of Iba1 ten times stronger in the lesion compared to rats without a spinal cord injury. However, the expression of anti-Iba1 increased only fourfold in the experimental animals given BMSCs, demonstrating the benefits of stem cell administration [54]. The MAPK signaling pathway is directly activated following microglial activation, triggering long-term potentiation and central sensitization in pain mechanisms [47].

**Figure 2.**
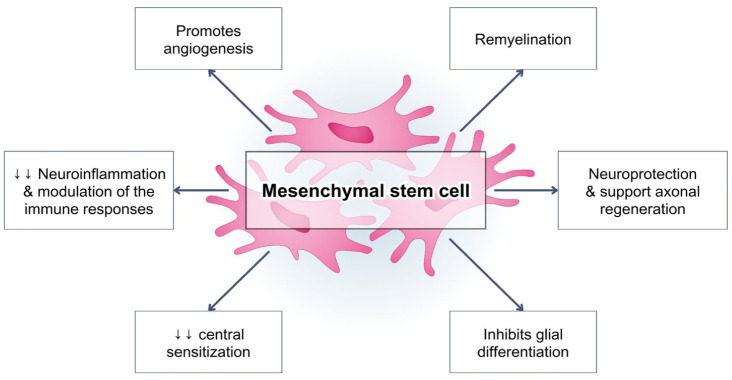
The role of Mesenchymal stem cells (MSCs) in neuropathic pain mechanism. MSCs can modulate neuroinflammation and immune cell responses, promote angiogenesis, neuroprotective effect, sustain axonal regeneration, remyelination through their paracrine role of secreting exosomes/microvesicles and inhibits glial cell differentiation.

### 2.8. Mesenchymal stem cells potential side effects

The problem of unfavorable events requires serious attention. Some of them may be due to infection, despite cell therapies being ineffective. MSCs resulted in side effects in 12% of COVID-19 patients after treatment. COVID-19 treatment with MSCs has been criticized for potentially causing blood clotting issues due to the release of a procoagulant tissue factor [55]. Common complications of severe pneumonia include liver damage, heart failure, and an allergic rash [56]. Overdosing of cell treatments is more likely to cause kidney damage [57]. MSC therapy may cause pneumonic embolism [58]. The risks of thromboembolization and the effectiveness of the cell treatment depend on the beneficiary's medical history and phenotyping highlights [59]. Večerić-Haler et al. described a clinical case of capillary spill disorder mimicking with severe kidney failure after autologous MSCs transplantation in a patient with previous lymphoblastic leukemia [60]. Despite this, we should differentiate these cases from other adverse events that occurred due to the poor control of MSC separation. Recently, a patient with chronic kidney disease experienced interstitial tissue fibrosis and tubular decay after receiving autologous adipose-derived MSCs [61]. In a study, 2372 patients with a degenerative joint disease were treated with autologous MSCs infusions [62]. Most adverse events were mild post-procedure pain or complications of the disease. Despite this, neoplasms and neurological and vascular signs were serious adverse events. Neurologic and vascular events were found in 6 and 5 cases, respectively, representing 0.25% and 0.21% of the population, respectively. The systemic administration of MSCs causes adverse events due to their immunosuppressive properties. Specifically, MSCs treatment increases the risk of pneumonia-related death after HSC transplantation [63]. Additionally, stem cell transplantation is linked to alterations in various lymphocyte populations (CD4+ T-helper cells, CD8+ T-cells, CD19+, and CD20+ B-lymphocytes). The CD4/CD8 ratio and CD19+ and CD20+ cell populations decreased in stem cell-treated patients [64]. Most studies described the safe use of MSCs with minor side effects. The most common example was fever, which occured in 22% of patients following hUC-MSC mixture for COVID-19 treatment [65], in 9.8% of patients after hUC-MSCs treatment for Crohn's disease [66], and in 85% of patients with active multiple sclerosis after autologous BM-MSCs treatment [67]. Using allogeneic MSCs to treat liver failure resulted in fever in 19.2% of patients during 5–24 weeks of follow-up. Fever was linked to the phosphate-buffered saline (PBS) buffer. A meta-analysis revealed a connection between MSCs and temporary fever. Intra-articular infusions of MSCs lessened pain in patients within 48–72 hours [68].

## 3 Conclusion

Neuropathic pain is induced by various mechanical damages and through a complex cascade resulting from a nerve injury, and is manifested as heterotopic pain, hyperalgesia, burning sensations, tingling, and spontaneous pain. After a nerve injury, neuroinflammatory processes and tissue repair can lead to a state of hyperexcitability known as peripheral sensitization. This condition often resolves as a part of the healing process and subsiding inflammation. The CNS refers to functional synaptic plasticity that depends on the activity and leads to pain hypersensitivity. MSCs have unique activities, including peripheral and spinal mechanisms, and serves as an inflammation protector by enhancing various anti-inflammatory molecules, thereby suppressing the release of pro-inflammatory cytokines, particularly reducing various inflammatory mediators. The activation of GFAP and protein induction plays a crucial role in astrogliosis after a nerve injury and axonal degeneration in non-myelinating Schwann cells, as characterized by an increased GFAP expression in an axonal neuropathy. MSCs can suppress astrocytes activation through GFAP expression following damage to the somatosensory system and may enhance GDNF expression. Managing neuropathic pain poses its own challenges due to its complex pathomechanism that involves the entire nervous system, both the CNS and the PNS. However, stem cell therapy has a strong therapeutic potential and is quite promising.
